# Early Prediction of Hepatic Decompensation in Cirrhosis Using Optimised XGBoost Models at the Initial Outpatient Hepatology Visit

**DOI:** 10.1111/liv.70560

**Published:** 2026-02-23

**Authors:** Micah Grubert Van Iderstine, Sem Perez, Gregory S. Jackson, Tyler Szun, Caelan Stephanson, Megan Weisshaar, Gerald Y. Minuk, Nabiha Faisal

**Affiliations:** ^1^ Max Rady College of Medicine University of Manitoba Winnipeg Manitoba Canada; ^2^ Section of Hepatology, Department of Internal Medicine, Max Rady College of Medicine University of Manitoba Winnipeg Manitoba Canada

**Keywords:** cirrhosis, decompensation, machine learning, outpatient hepatology, prognostic modelling, risk prediction

## Abstract

**Background and Aims:**

Hepatic decompensation represents a critical transition in cirrhosis, leading to increased morbidity, mortality and healthcare utilisation. Identifying patients at risk of decompensation remains a clinical challenge. We aimed to develop and validate XGBoost models to predict hepatic decompensation at multiple time points using clinical data available at a patient's initial outpatient hepatology visit.

**Methods:**

We conducted a retrospective cohort study including 2208 adult patients with cirrhosis or its complications seen in hepatology clinics between 1985 and 2022. Patients were classified as compensated or decompensated based on a keyword search of the Philip and Ellie Kives Clinical Database, with decompensation dates confirmed by chart review. Sixteen routinely available variables including demographics, biochemical parameters and disease aetiology were used as predictors. Logistic regression and XGBoost models were trained to predict hepatic decompensation at 1, 3, 5 and 10 years, with a random 20% holdout test set used for validation. XGBoost models were tuned to optimise the precision‐recall area under the curve (PR‐AUC). Performance was evaluated using AUROC, precision, recall and F1 scores.

**Results:**

XGBoost models outperformed logistic regression at most time points, demonstrating strong performance at 3 and 5 years. Recall was 0.42, 0.98, 0.98 and 0.82 at 1, 3, 5 and 10 years respectively. Corresponding AUROC values were 0.85, 0.88, 0.81 and 0.89.

**Conclusions:**

Optimised XGBoost models demonstrated robust predictive accuracy for medium‐ and long‐term hepatic decompensation among patients with compensated cirrhosis. These models may support early risk stratification and enable personalised management strategies to prevent clinical deterioration.

## Key Points

- XGBoost models were developed using routinely available baseline clinical data to predict hepatic decompensation at 1, 3, 5 and 10 years in patients with compensated cirrhosis.
- In a cohort of 2208 patients, XGBoost models predicted hepatic decompensation with good accuracy, demonstrating AUROC > 0.80 and recall of 0.98 at 3 and 5 years.
- Key predictors included platelet count, albumin, INR, bilirubin, age and disease aetiology.
- Machine learning models optimised for Precision‐Recall AUC (PR‐AUC) may enable risk stratification that supports earlier intervention and personalised care for patients with cirrhosis.

**Abbreviations**
ACE: AI‐cirrhosis‐ECG
ALBI‐FIB4: albumin‐bilirubin fibrosis‐4
ALD: alcohol‐related liver disease
ALP: alkaline phosphatase
ALT: alanine aminotransferase
AST: aspartate aminotransferase
AUROC: area under the receiver operating characteristic curve
EPOD: early prognostic scoring system of clinical decompensation
GGT: gamma‐glutamyl transferase
HCC: hepatocellular carcinoma
INR: international normalised ratio
MASH: metabolic‐dysfunction associated steatohepatitis
MELD: model for end‐stage liver disease
ML: machine learning
PR‐AUC: precision‐recall area under the curve
SHAP: SHapley Additive exPlanations

## 1 Introduction

Cirrhosis represents the end stage of all chronic liver diseases and is characterised by progressive hepatic fibrosis, declining liver function and the emergence of life‐threatening complications. This disease trajectory is associated with substantial morbidity, increased mortality and diminished quality of life. Clinically, cirrhosis is categorised into two stages: compensated (asymptomatic) and decompensated (symptomatic). Decompensation is defined by the onset of ascites, variceal bleeding, hepatic encephalopathy or jaundice, and marks a critical turning point in disease progression. The annual incidence of decompensation in compensated cirrhosis is estimated at 5%–7%, with 1‐year mortality rising from approximately 5% in compensated individuals to nearly 20% following decompensation [1, 2]. Timely identification of patients at high risk for decompensation is therefore essential for guiding enhanced surveillance, early intervention and individualised management strategies aimed at improving long‐term outcomes.

Recent advances in machine learning (ML) have generated interest in its application to hepatology, particularly for risk prediction and clinical decision support. ML models utilising structured clinical data including laboratory values, imaging, histopathology and genomic information have been successfully applied to several domains in hepatology such as non‐invasive assessment of liver fibrosis, classification of focal liver lesions and post‐transplant graft survival prediction [3, 4, 5, 6]. Despite this progress, the application of ML to predict hepatic decompensation remains limited and underdeveloped. Most existing models are hampered by methodological challenges, including small training datasets, inadequate external validation, limited generalisability and insufficient reporting of critical performance metrics such as class specific precision, recall and F1 scores. These limitations reduce transparency and hinder clinical translation. Furthermore, few models offer actionable, time‐sensitive risk estimates to support clinical decision‐making in real‐world settings.

A major limitation of prior work is the continued reliance on traditional clinical scoring systems such as Model for End‐stage Liver Disease (MELD), Child‐Pugh and Albumin‐Bilirubin Fibrosis‐4 (ALBI‐FIB4) scores which, although widely used to assess liver disease severity, provide only coarse estimates of risk and lack the flexibility required for dynamic, individualised predictions [7]. In 2022, the Early Prognostic scoring system Of clinical Decompensation (EPOD) was introduced as an attempt to address these shortcomings using a simplified algorithm based on platelet count, albumin and bilirubin. While EPOD demonstrated improved accuracy in predicting 3‐year decompensation compared to MELD and Child‐Pugh scores, it still falls short of clinical utility, particularly in real‐time or patient‐specific decision‐making contexts [8]. Nonetheless, these studies have consistently identified variables such as patient age, platelet count and liver function tests as relevant predictors. Additionally, growing evidence suggests that the risk and clinical course of decompensation differ by underlying disease aetiology, emphasising the need for aetiology‐specific modelling approaches [9].

Emerging deep learning‐based approaches have shown early promise. For example, a recent study by Ahn et al. [10] has shown that the AI‐Cirrhosis‐ECG (ACE) score based on electrocardiogram tracings can predict hepatic decompensation risk with high accuracy. However, the model's reliance on serial recalculation at each visit, its lack of time‐specific risk estimates and performance degradation in external validation cohorts highlight ongoing challenges related to scalability and generalisability. Other exploratory models incorporating genetic and biochemical data using Support Vector Machines and Random Forest modelling have shown modest predictive performance [11]. However, small sample sizes and lack of sensitivity reporting limit interpretability and clinical translation.

Extreme Gradient Boosting (XGBoost), a powerful decision tree‐based ensemble learning method, has emerged as a leading algorithm for structured clinical data. Its advantages include high predictive performance in binary classification, robustness to missing data and its ability to minimise overfitting [12, 13]. Despite its widespread adoption across clinical prediction tasks, there are no studies that successfully use tuned XGBoost models for the prediction of hepatic decompensation to date.

In this study, we aim to develop and validate a highly optimised XGBoost‐based ML model to predict hepatic decompensation at multiple clinically relevant time points using routinely collected clinical data from patients with compensated cirrhosis at their initial outpatient hepatology consultation. Our goal is to enable individualised, time‐sensitive risk stratification to support earlier intervention, enhanced monitoring and the implementation of personalised care pathways to prevent or delay hepatic decompensation.

## 2 Methods

This retrospective cohort study was approved by the Human Research Ethics Board at University of Manitoba Bannatyne Campus (HS26114), and the Manitoba Provincial Health Research Privacy Committee (P2024‐22). Patient informed consent was waived by the respective ethics committees due to the retrospective nature of the study.

### 2.1 Study Population

Patients were included if they had been evaluated in an outpatient hepatology clinic at the University of Manitoba between 1985 and 2022 and documented diagnosis of cirrhosis recorded in the Philip and Ellie Kives Clinical Database. Cirrhosis was diagnosed by a hepatologist based on liver biopsy, transient elastography (fibroscan) or cross‐sectional imaging findings consistent with cirrhosis. To be included in the analysis, patients were required to have a complete panel of laboratory investigations performed at or within 3–6 months prior to their initial outpatient hepatology visit. This included alanine aminotransferase (ALT), aspartate aminotransferase (AST), gamma‐glutamyl transferase (GGT), alkaline phosphatase (ALP), international normalised ratio (INR), total bilirubin, platelet count, serum creatinine, serum albumin, serum sodium and ferritin. Liver disease aetiology was manually extracted from clinical records and encoded as binary variables to account for patients with single or overlapping causes of cirrhosis. This processing strategy allowed for multiple disease etiologies to be recorded for individual patients while maintaining a single outcome per study ID, thereby preventing double counting of events in the predictive models. Demographic data included age, sex and residential postal code, which was subsequently mapped to urban or rural designation, with urban status defined as living in a population centre with > 50 000 residents. Patients were categorised into four longitudinal cohorts with follow‐up of a minimum of 1, 3, 5 and 10 years. Patients who developed hepatic decompensation were included even if they were later lost to follow‐up. Patients who developed decompensated cirrhosis were identified through a structured keyword search of the Philip and Ellie Kives Clinical Database. Search terms included ‘decompensated cirrhosis’, ‘ascites’, ‘hepatic encephalopathy’, ‘hepatorenal syndrome’, ‘hyperbilirubinemia’, ‘jaundice’ ‘variceal bleeding’, ‘liver transplant’, ‘hepatocellular carcinoma’, and relevant medications (e.g., lactulose, rifaximin, furosemide, spironolactone, tacrolimus, mycophenolate mofetil, sirolimus). The terms ‘liver transplant’, ‘tacrolimus’, ‘mycophenolate mofetil’ and ‘sirolimus’ were included to capture patients with cirrhosis undergoing liver transplantation for non‐acute liver failure. All flagged records underwent manual chart review to confirm the occurrence and timing of the first decompensation event, as well as its associated clinical manifestations. To ensure capture of true first decompensation events, charts and hospital admissions from a minimum of 12 months prior to the first hepatology clinic visit were reviewed, and the initial consultation notes of the treating physician summarising long term symptom burden were reviewed to cover more extended periods.

Patients were excluded if, upon chart review, they were found to have decompensated prior to their first hepatology visit, had a diagnosis of paediatric liver disease managed prior to transition to adult care, had no confirmatory evidence of cirrhosis or lacked follow‐up. Patients with missing, incomplete or erroneous clinical data at their initial visit were also excluded.

### 2.2 Definition of Hepatic Decompensation

Hepatic decompensation was defined as the first documented occurrence of any of the following clinical events: ascites, jaundice, variceal haemorrhage, hepatic encephalopathy, hepatorenal syndrome, hepatopulmonary syndrome or hepatic hydrothorax as noted in the medical chart. In addition, patients without overt decompensation events who subsequently developed hepatocellular carcinoma (HCC) or underwent liver transplantation for non‐acute liver failure were also classified as having decompensated cirrhosis.

In cases where a discrete decompensation event could not be identified prior to HCC diagnosis or liver transplantation, a surrogate decompensation date was assigned as 6 months prior to the earliest of these two events. Manual chart review was performed for all suspected cases to confirm the occurrence and timing of decompensation, ensuring accurate classification and date assignment.

To improve the reliability of decompensation date assignment in ambiguous cases (e.g., patients with limited documentation or concurrent complications), two independent reviewers blindly assessed the clinical records, with discrepancies resolved by consensus or involvement of a senior hepatologist.

### 2.3 XGBoost Modelling

Model inputs included patient demographics, liver disease aetiology and laboratory parameters obtained at the time of the initial outpatient hepatology clinic visit, as summarised in Tables 1 and 2.

**TABLE 1:** Distribution of patient liver disease etiologies and compensation status at 1, 3, 5 and 10 years of follow‐up.

Minimum follow‐up	1 year		3 years		5 years		10 years	
Patient status	Compensated *N* (%)	Decompensated *N* (%)	Compensated *N* (%)	Decompensated *N* (%)	Compensated *N* (%)	Decompensated *N* (%)	Compensated *N* (%)	Decompensated *N* (%)
Disease aetiology^a^								
Total *N*	2103	105	1730	196	1360	259	732	351
MASH	804 (38.2%)	22 (21.0%)	676 (39.1%)	46 (23.5%)	502 (36.9%)	64 (24.7%)	215 (29.4%)	84 (23.9%)
HCV	419 (19.9%)	11 (10.5%)	350 (20.2%)	25 (12.8%)	295 (21.7%)	35 (13.5%)	192 (26.2%)	58 (16.5%)
ALD	271 (12.9%)	40 (38.1%)	205 (11.8%)	62 (31.6%)	153 (11.2%)	73 (28.2%)	64 (8.7%)	91 (25.9%)
PBC	273 (13.0%)	5 (4.8%)	251 (14.5%)	15 (7.7%)	212 (15.6%)	21 (8.1%)	137 (18.7%)	27 (7.7%)
Unknown	178 (8.5%)	7 (6.7%)	111 (6.4%)	13 (6.6%)	72 (5.3%)	17 (6.6%)	34 (4.6%)	17 (4.8%)
HBV	145 (6.9%)	8 (7.6%)	137 (7.9%)	12 (6.1%)	122 (9.0%)	16 (6.2%)	83 (11.3%)	19 (5.4%)
AIH	138 (6.6%)	10 (9.5%)	127 (7.3%)	14 (7.1%)	108 (7.9%)	21 (8.1%)	62 (8.5%)	31 (8.8%)
PSC	89 (4.2%)	4 (3.8%)	78 (4.5%)	12 (6.1%)	66 (4.9%)	17 (6.6%)	44 (6.0%)	24 (6.8%)
Other	98 (4.7%)	10 (9.5%)	72 (4.2%)	21 (10.7%)	55 (4.0%)	24 (9.3%)	25 (3.4%)	40 (11.4%)

Abbreviations: AIH, autoimmune hepatitis; ALD, alcohol‐related liver disease; HBV, hepatitis B virus; HCV, hepatitis C virus; MASH, metabolic‐dysfunction associated steatohepatitis; PBC, primary biliary cholangitis; PSC, primary sclerosing cholangitis.

^**a**^ Disease aetiology is not mutually exclusive, with several patients classified as having overlapping liver disease processes.

**TABLE 2:** Baseline demographics and laboratory parameters at initial hepatology visit, stratified by clinical status at 1, 3, 5 and 10 years of follow‐up.

Minimum follow‐up	Reference range	1 year		3 years		5 years		10 years	
Patient status		Compensated	Decompensated	Compensated	Decompensated	Compensated	Decompensated	Compensated	Decompensated
Total *N*		2103	105	1730	196	1360	259	732	351
Sex (F)		1094 (52.0%)	53 (50.5%)	924 (53.4%)	97 (49.5%)	736 (54.1%)	129 (49.8%)	398 (54.4%)	185 (52.7%)
Rural		792 (37.7%)	45 (42.9%)	636 (36.8%)	80 (40.8%)	496 (36.5%)	102 (39.4%)	252 (34.4%)	136 (38.7%)
Age at first lab		52.22 ± 13.05	55.33 ± 14.59	51.25 ± 12.76	55.41 ± 14.45	50.49 ± 12.54	54.58 ± 14.25	48.28 ± 11.88	53.24 ± 14.23
Length of follow‐up (yrs)		8.71 ± 6.66	6.42 ± 6.40	10.30 ± 6.39	7.18 ± 6.95	11.96 ± 6.15	8.01 ± 7.31	15.90 ± 5.66	9.37 ± 7.57
ALT (IU/L)	17–36	102.08 ± 197.75	69.71 ± 108.63	108.48 ± 211.16	81.30 ± 152.71	115.86 ± 226.02	89.17 ± 192.08	132.51 ± 257.90	97.15 ± 183.67
AST (IU/L)	15–37	90.53 ± 183.34	92.96 ± 114.24	93.44 ± 196.18	98.48 ± 129.35	98.66 ± 214.25	98.98 ± 129.79	111.94 ± 253.27	105.23 ± 160.17
ALP (IU/L)	50–136	168.75 ± 190.36	189.48 ± 200.74	168.05 ± 192.84	219.47 ± 242.78	172.07 ± 195.43	211.35 ± 237.45	179.02 ± 198.28	206.11 ± 231.11
GGT (IU/L)	15–85 (M), 5–55 (F)	220.13 ± 325.84	185.30 ± 255.93	217.43 ± 315.79	218.09 ± 284.10	223.55 ± 320.02	221.96 ± 280.89	226.87 ± 302.39	246.97 ± 343.62
Total bilirubin (μmol/L)	3–17	19.57 ± 38.85	40.48 ± 48.46	18.70 ± 38.07	37.45 ± 43.96	18.70 ± 38.78	34.06 ± 41.05	20.75 ± 48.58	31.46 ± 39.09
Albumin (g/L)	34–50	37.47 ± 6.43	30.66 ± 7.65	38.03 ± 6.27	31.64 ± 7.39	38.30 ± 6.41	32.56 ± 7.15	38.49 ± 6.66	33.72 ± 6.89
Creatinine (μmol/L)	49–93 (M), 22–75 (F)	81.40 ± 124.54	84.12 ± 70.49	81.90 ± 135.76	76.97 ± 53.66	78.82 ± 61.92	74.41 ± 47.69	74.03 ± 32.35	75.54 ± 57.00
Sodium (mmol/L)	136–146	139.23 ± 6.20	137.70 ± 4.83	139.34 ± 6.60	137.99 ± 4.61	139.55 ± 6.19	138.28 ± 4.34	139.47 ± 7.87	138.34 ± 4.20
Platelets (×10^9^/L)	130–380	199.80 ± 90.86	153.90 ± 102.82	204.49 ± 89.65	150.80 ± 97.29	207.58 ± 89.54	150.83 ± 93.72	216.17 ± 89.00	152.15 ± 90.05
INR	1.0	1.11 ± 0.34	1.32 ± 0.33	1.10 ± 0.35	1.29 ± 0.38	1.10 ± 0.37	1.27 ± 0.38	1.10 ± 0.46	1.24 ± 0.35
Ferritin (μg/L)	24–336 (M), 11–307 (F)	276.51 ± 492.21	325.26 ± 490.14	276.24 ± 506.98	291.27 ± 418.80	270.25 ± 479.25	310.72 ± 631.63	273.73 ± 561.70	282.47 ± 556.50

Abbreviations: ALP, alkaline phosphatase; ALT, alanine aminotransferase; AST, aspartate aminotransferase; GGT, gamma‐glutamyl transferase; INR, international normalised ratio.

XGBoost Models were developed to predict hepatic decompensation at four distinct future time points: 1, 3, 5 and 10 years. For each time‐specific model, a random search across a broad hyperparameter space was conducted to identify optimal configurations. Hyperparameters were selected to maximise the area under the precision recall curve (PR‐AUC), which prioritises accurate prediction of the positive class (i.e., patients who develop decompensation), particularly in imbalanced datasets. This approach was preferred over optimising the harmonic mean accuracy of the model (F1) due to a relatively low proportion of decompensation events, and the clinical imperative to minimise false negatives. These distinct sets of hyperparameters were optimised across 1000 model iterations with 5‐fold cross validation applied during training to mitigate overfitting and enhance generalisability. Features were scaled using a standard scaler, after a random 20% holdout sample was reserved for final model validation and performance assessment on unseen clinical data. Model performance was evaluated using class specific model metrics of precision, recall, F1, and as well as the macro‐averaged values of these metrics and the area under the receiver operating characteristic curve (AUROC) [14]. To support interpretability receiver operating characteristic curves, test sample class prediction graphs, and SHapley Additive exPlanations (SHAP) values were generated for each prediction window [15].

All data processing, model training and visualisation was performed using Python (v3.11.3), with key libraries including XGBoost, matplotlib, seaborn, SciKitLearn and SHAP.

### 2.4 Logistic Regression Modelling

Logistic regression models were developed as baseline comparators using the same model inputs and time points as the XGBoost models. Model performance was evaluated using the identical metrics and visualisation tools. Decision thresholds were chosen at each of the time points to optimise F1 score. Attempts to optimise for decompensation recall led to poor overall model performance, highlighting the challenge of balancing sensitivity and precision when there are fewer cases in the dataset.

### 2.5 Descriptive Statistics

Descriptive statistics were generated to characterise the study population by decompensation status at 1, 3, 5 and 10 years follow‐up intervals. Counts were shown for categorical variables, and continuous variables were represented by the mean and standard deviation where applicable.

## 3 Results

**FIGURE 1:**
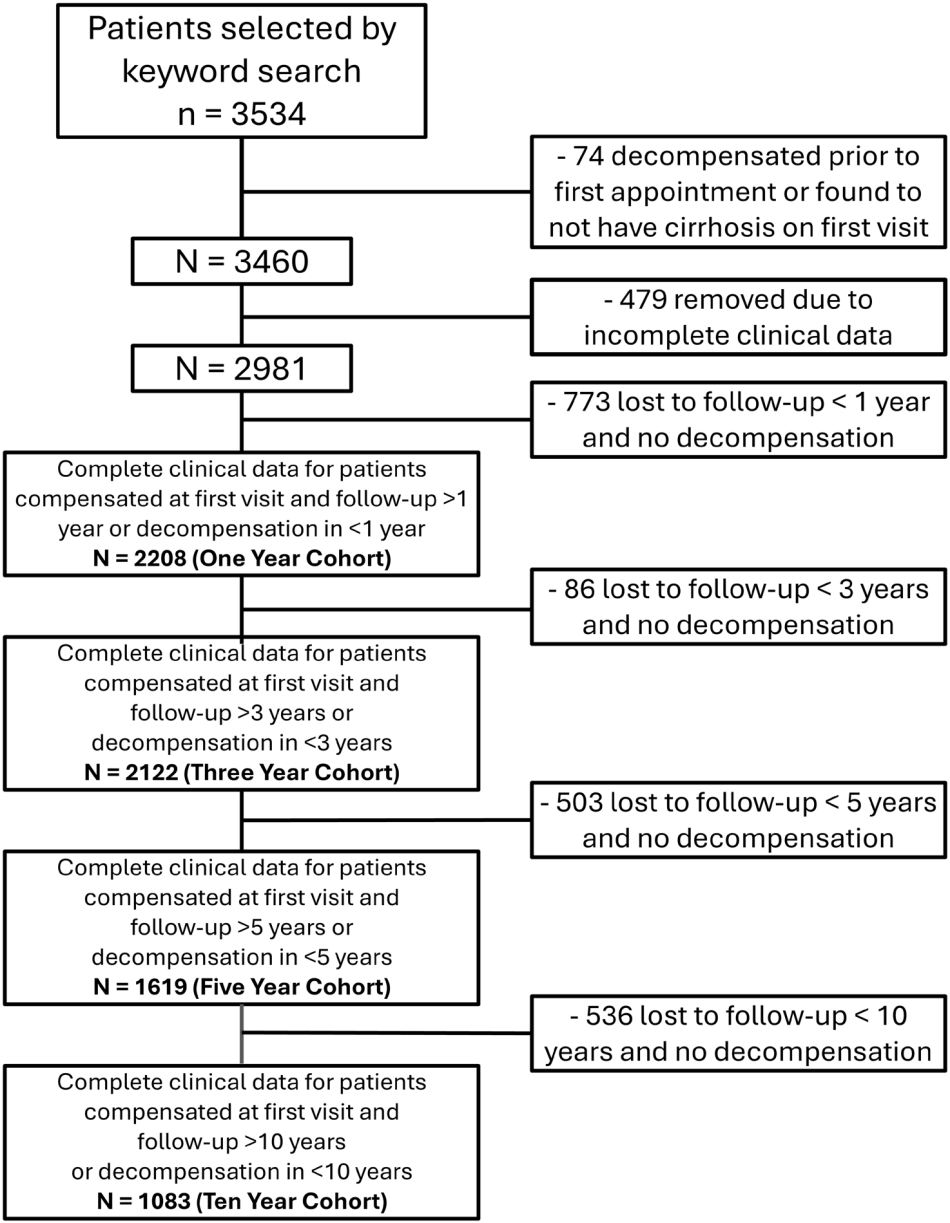
Flow chart demonstrating patient selection process. The bold text reflects the final study populations of the four cohorts included in the analysis after excluding ineligible patients.

There were 2208 patients that met the eligibility criteria and either had a minimum follow‐up of 1 year or became decompensated within that interval (Figure 1). At the 1‐year mark, 2103 patients remained compensated (Table 1). The mean age of compensated patients was 52.2 ± 13.1 years, and their average follow‐up duration from their initial outpatient hepatology visit was 8.7 ± 6.7 years. Of these patients, 52.0% were female and 37.7% resided in rural municipalities.

One hundred five patients experienced decompensation within the first year. Their mean age was 55.3 ± 14.6 years and the average follow‐up duration following their first outpatient hepatology visit was 6.4 ± 6.4 years. Among them, 50.5% were female and 42.9% were from rural municipalities. Baseline laboratory values for both groups are summarised Table 1.

Descriptive characteristics, including demographics and laboratory data stratified by decompensation status at 3, 5 and 10‐year follow‐up intervals are also presented in Table 1. A total of 1083 patients were followed for at least 10 years or decompensated within that period. Out of the 351 decompensated patients, 261 patients had symptomatic decompensation, 37 were classified as decompensated due to a diagnosis of HCC and 53 were classified as decompensated due to liver transplantation.

Table 2 presents the distribution of liver disease etiologies and corresponding decompensation status across all time points. Metabolic‐Dysfunction Associated Steatohepatitis (MASH) was the most prevalent aetiology among the compensated group at 1 year (*n* = 804), while Alcohol‐related Liver Disease (ALD) was the leading cause among those who decompensated within the same interval (*n* = 40).

Performance metrics for both logistic regression and XGBoost models are summarised in Table 3. On the 20% holdout test set, logistic regression models achieved decompensation recall of 0.32, 0.90, 0.76 and 0.80 at 1, 3, 5 and 10 years respectively. Corresponding AUROC values were 0.84, 0.87, 0.85 and 0.84 at their respective time intervals.

**TABLE 3:** Performance metrics for logistic regression and XGBoost models predicting hepatic decompensation at 1, 3, 5 and 10 year intervals.

Prediction interval	Outcome	Precision	Recall	F1	AUROC	PR‐AUC	Support^a^
Logistic regression							
1 year	Decompensation	0.43	0.32	0.36			19
1 year	Compensated	0.97	0.98	0.98			423
1 year	Average	0.7	0.65	0.67	0.841	0.219	442
XGBoost							
1 year	Decompensation	0.17	0.53	0.26			19
1 year	Compensated	0.98	0.89	0.93			423
1 year	Average	0.57	0.71	0.59	0.851	0.274	442
Logistic regression							
3 years	Decompensation	0.37	0.9	0.52			50
3 years	Compensated	0.98	0.77	0.86			336
3 years	Average	0.67	0.83	0.69	0.868	0.470	386
XGBoost							
3 years	Decompensation	0.23	0.98	0.37			50
3 years	Compensated	0.99	0.5	0.66			336
3 years	Average	0.61	0.74	0.51	0.880	0.460	386
Logistic regression							
5 years	Decompensation	0.39	0.76	0.52			49
5 years	Compensated	0.95	0.79	0.86			275
5 years	Average	0.67	0.77	0.69	0.847	0.514	324
XGBoost							
5 years	Decompensation	0.19	0.98	0.31			49
5 years	Compensated	0.99	0.24	0.39			275
5 years	Average	0.59	0.61	0.35	0.806	0.457	324
Logistic regression							
10 year	Decompensation	0.66	0.8	0.72			71
10 year	Compensated	0.89	0.79	0.84			146
10 year	Average	0.77	0.8	0.78	0.843	0.714	217
XGBoost							
10 year	Decompensation	0.71	0.82	0.76			71
10 year	Compensated	0.9	0.84	0.87			146
10 year	Average	0.81	0.83	0.81	0.887	0.795	217

^a^ Denotes the number of instances of the indicated patient status were present in the random 20% test sample used for model validation.

**FIGURE 2:**
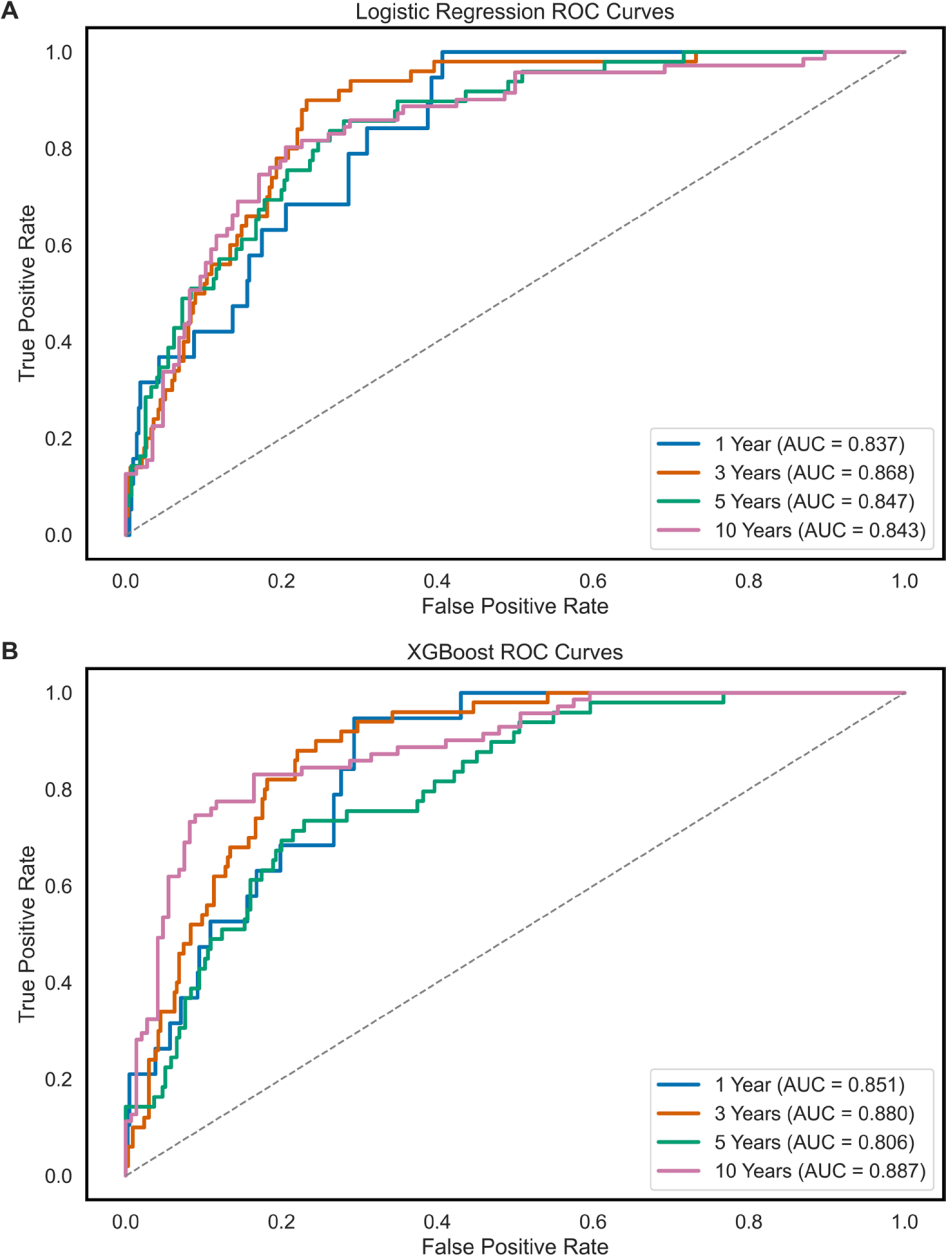
Receiver operating characteristic (ROC) curves for logistic regression and XGBoost models predicting hepatic decompensation at four defined timepoints. ROC curves for the logistic regression series of models are shown in Panel A, and the ROC curves for the XGBoost series of models are shown in Panel B, with each evaluation timepoint highlighted by colour.

**FIGURE 3:**
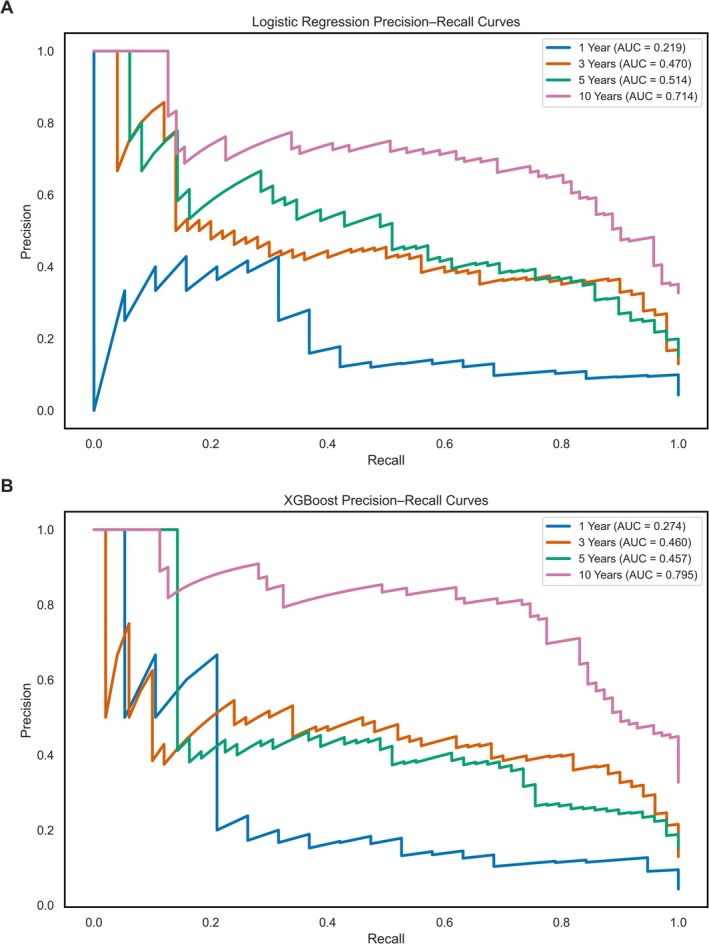
Precision‐Recall (PR) curves for logistic regression and XGBoost models predicting hepatic decompensation at four defined timepoints. PR curves for the logistic regression series of models are shown in Panel A, and the PR curves for the XGBoost series of models are shown in Panel B, with each evaluation timepoint highlighted by colour.

**FIGURE 4:**
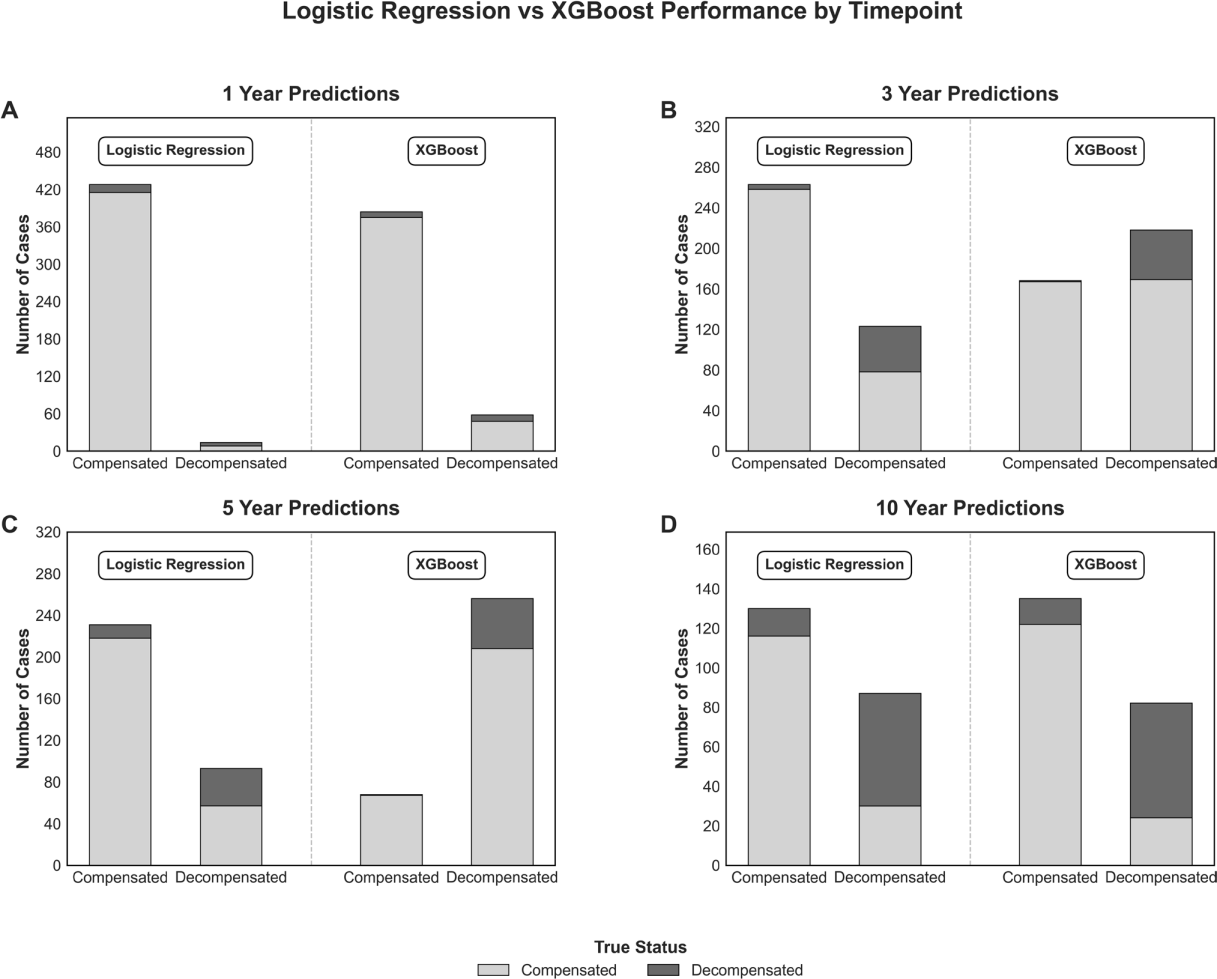
Comparison of model predictions and true patient status on a 20% test sample of each timepoint's dataset. Panels 4A–D represent the predictions by logistic regression and XGBoost models across the 1, 3, 5 and 10 year datasets, with true patient status represented by colour.

**FIGURE 5:**
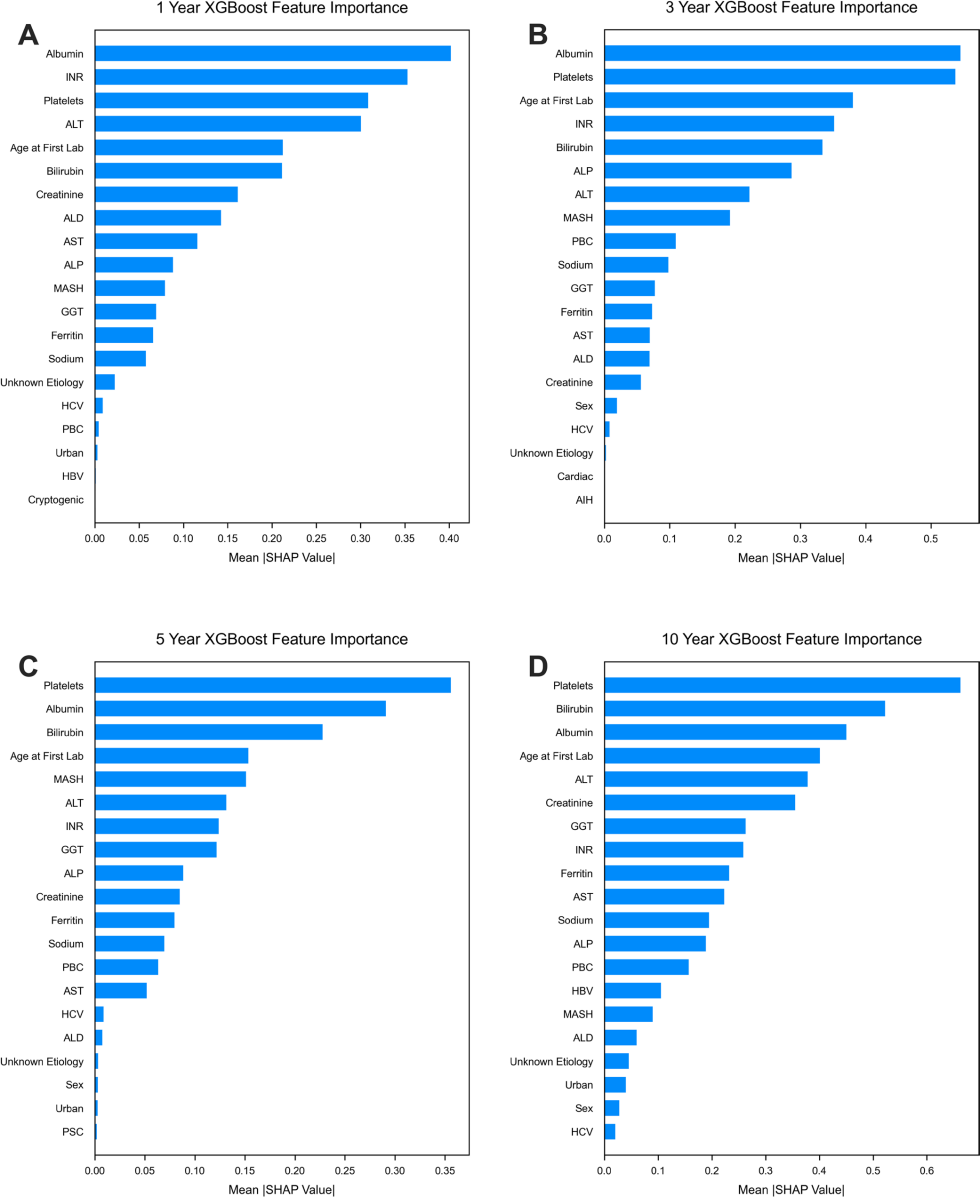
Model feature importance evaluated by SHapley Additive exPlanations (SHAP) values. SHAP values are presented for four XGBoost models for predicting hepatic decompensation at 1 (A), 3 (B), 5 (C) and 10 (D) years to demonstrate feature importance in model decision making.

XGBoost models achieved decompensation recall values of 0.53, 0.98, 0.98 and 0.82 across the same time points with AUROC values of 0.85, 0.88, 0.81 and 0.89, respectively. Model discrimination is visualised via ROC curves in Figure 2. PR‐AUC curves are presented in Figure 3. Class prediction distributions for each model are presented as bar plots in Figure 4, and XGBoost model SHAP values are shown in Figure 5.

## 4 Conclusions

This study demonstrates that optimised XGBoost models can effectively predict hepatic decompensation at 3‐, 5‐ and 10‐year intervals using routinely available clinical data from a patient's initial outpatient hepatology visit (Figure 2, Table 3). Model performance was consistently strong, with AUROC values exceeding 0.80 at all time points and recall values reaching 0.98 at both 3 and 5 years prediction windows. These results markedly outperform traditional prognostic models such as MELD and FIB‐4, which typically yield AUROCs between 0.60 and 0.75 for similar time periods [7, 8]. Unlike conventional scoring systems, which are static and optimised for short‐term mortality or fibrosis staging, XGBoost models capture complex, nonlinear interactions between clinical variables and accommodate time‐dependent predictions [13]. This dynamic modelling capability makes it more suitable for individualised, prospective risk stratification in patients with compensated cirrhosis.

Interestingly, with an optimal decision threshold set, logistic regression models also performed well. While they showed slightly lower AUROC values at 1, 3 and 10 years, they marginally outperformed the XGBoost model at 5 years, likely reflecting that models' tendency to overpredict decompensation during this interval (Table 3). Importantly, the logistic regression models excel in identifying patients from the larger compensated group rather than the smaller decompensated group and also showed higher F1 scores. The strong performance of logistic regression models in prediction emphasises the strong linear relationships between the variables that comprise the MELD score and patient outcomes, and underscores the value of adding variables to this widely used scoring model, which has historically performed poorly in predicting decompensation episodes [11]. For consistency, all XGBoost models in this study were optimised for PR‐AUC to prioritise recall, given the clinical imperative to minimise false negatives (Figure 3). Although a less stringent model optimised for F1 score may have been able to predict decompensation episodes in the 5‐year cohort with a smaller drop in precision, and it would be of value to explore this in a future study.

The model performance by PR‐AUC shows logistic regression models obtain better precision‐recall discrimination in the 3‐ and 5‐year cohorts, however in the 1‐ and 10‐year cohorts, XGBoost models show an advantage in PR‐AUC (Table 3). This is particularly notable in the 1‐year cohort, as prediction over this interval was less effective across both models (recall: 0.53 for XGBoost, 0.32 for logistic regression), possibly reflecting clinical instability in newly referred patients. A substantial proportion of early decompensations (38%) occurred in patients with ALD, a group known for unpredictable and rapid deterioration (Table 1). This is supported by the model's SHAP values, which show a diagnosis of ALD to be a key driver of early decompensation predictions in the 1‐year cohort model (Figure 5). These findings are consistent with prior population‐based cohort studies showing higher early decompensation risk in ALD relative to MASH [16]. However, in our cohort decompensation rates at both 1 year and 5 years were higher in ALD patients, suggesting potential lead‐time or referral bias, where ALD patients may be referred later in their disease course. This pattern aligns with a prior systematic review from 2019, reporting higher 5‐year decompensation rates in ALD compared to those in MASH cirrhosis [9].

MASH also emerged as a strong predictor of decompensation across all time points, with a particularly high SHAP score at 5 years (Figure 5). This finding reflects shifting epidemiology, as MASH has now become the leading cause for liver‐related referrals in North America [17, 18]. The predominance of MASH in our cohort reinforces the contemporary relevance of our models for tertiary care populations.

The most impactful variables across each of the XGBoost models were the markers for liver function (platelets, albumin, INR and bilirubin) [7, 9, 11]. Notably, INR was the strongest predictor at 1‐year, highlighting the importance of coagulopathy as a risk factor for acute decompensation. This observation is consistent with studies identifying INR as an independent predictor of short‐term mortality and adverse outcomes in patients with cirrhosis [19, 20]. Over longer prediction intervals, bilirubin and patient age became more predictive, with age overtaking INR in the 5 and 10 year models (Figure 5). The emergence of age as a predictor over time is consistent with literature showing higher decompensation risk in older individuals with cirrhosis [9, 21]. Other patient demographics (sex and urban vs. rural residence), aside from patient age, appeared to play a minimal role in influencing model predictions (Figure 5), suggesting that clinical and biochemical variables are the most powerful determinants of risk in this population.

Other laboratory markers such as ALT, creatinine, sodium and ferritin were scattered in importance across the models. Notably ALT was a consistent predictor for decompensation events, with lower levels influencing the models' decisions. This is consistent with the phenomenon of low levels of ALT activity seen in more advanced disease states [22, 23]. In general, laboratory values tended to influence the models to a greater extent than disease etiologies, aside from MASH and ALD. Primary biliary cholangitis also rose in importance in the longer time interval models, likely owing to the indolent nature of the disease and longer time course to decompensation than other liver diseases. Viral hepatitis similarly increased in importance in the 3, 5 and 10 year cohorts, which may reflect our inclusion of HCC as a sign of decompensation. Our results confirm previous findings that liver disease aetiology is an important determinant of decompensation risk and should be incorporated into predictive models [9].

A major strength of this study is the modelling strategy, which prioritised recall by optimising the PR‐AUC, enabling clear risk stratification and capturing nearly all patients who decompensated—an approach well‐aligned with clinical goals of early intervention, surveillance and transplant evaluation (Figure 4). The use of a large, real‐world cohort across a provincial health system enhances generalisability, while limiting model inputs to data from the initial outpatient visit improves feasibility for clinical integration. Manual chart review strengthened outcome classification and SHAP analysis provided interpretable insights into feature importance. The inclusion of logistic regression as a comparator highlights that while machine learning offers strong predictive power, simpler models applied to structured clinical data can still perform effectively. Future research may aim to explore alternative model optimisation strategies or dynamic recalibration to balance sensitivity with specificity.

Nonetheless, important limitations must be acknowledged. This was a single‐centre retrospective study, and external validation in independent cohorts is needed to confirm generalisability. Our cohort spans over three decades, during which diagnostic and management practices evolved. As with many longitudinal studies, survivorship bias must be acknowledged. This study included all decompensated patients regardless of length of follow‐up; however, patients who did not decompensate and were lost to follow‐up prior to their cohort's time window were removed from that cohort. This may have led to a higher proportion of patients with indolent disease processes included in the longer‐term well compensated cohorts. Patient ethnicity was not captured within this study, limiting evaluation of potential disparities. Also, the risk of decompensation was not assessed for generalised clinical categories (i.e., hepatocellular vs. cholestatic disease). The dataset, though large for clinical research, remains modest by machine learning standards, and surrogate decompensation dates were imputed in some cases, potentially introducing bias. Furthermore, while internal validation was robust, prospective validation and integration into clinical workflows will be necessary before real‐world implementation.

In conclusion, this study demonstrates the potential of machine learning, particularly optimised XGBoost modelling, to accurately predict hepatic decompensation using baseline clinical data. These models outperform traditional risk scores and offer interpretable, time‐specific predictions that may support earlier intervention and personalised care pathways. With further validation and prospective integration, such tools have the potential to transform risk stratification and improve outcomes for patients with compensated cirrhosis.

## Author Contributions

**Micah Grubert Van Iderstine:** conceptualisation, methodology, formal analysis, writing – original draft. **Sem Perez:** writing – original draft, data curation. **Gregory S. Jackson:** data curation, writing – review and editing. **Tyler Szun:** data curation. **Caelan Stephanson:** data curation. **Megan Weisshaar:** data curation. **Gerald Y. Minuk:** conceptualisation, writing – review and editing. **Nabiha Faisal:** conceptualisation, methodology, supervision, writing – review and editing.

## Funding

The authors have nothing to report.

## Ethics Statement

The research protocol was approved by the Human Research Ethics Board at University of Manitoba Bannatyne Campus (HS26114) and the Manitoba Provincial Health Research Privacy Committee (P2024‐22).

## Consent

Informed written consent from patients was waived by the Human Research Ethics Board due to the retrospective nature of the study.

## Conflicts of Interest

The authors declare no conflicts of interest.

## Data Availability

Clinical data is not available for distribution in accordance with our agreement with our respective ethics committees. The complete source code for this project is available upon request to the first author (Micah Grubert Van Iderstine, grubertm@myumanitoba.ca).
